# Mapping Peer Support in Antidepressant Discussions on Reddit: Pilot Network Analysis

**DOI:** 10.2196/85812

**Published:** 2026-06-12

**Authors:** Ejura Yetunde Salihu, Apoorva Reddy

**Affiliations:** 1 Social and Administrative Sciences Division, School of Pharmacy, University of Wisconsin-Madison, 610 N Whitney Way, Madison, WI, 53705, United States, 1 3095692413; 2 Department of Family Medicine and Community Health, University of Wisconsin-Madison, Madison, WI, United States; 3 Department of Surgery, University of Wisconsin-Madison, Madison, WI, United States

**Keywords:** antidepressant, side effects, withdrawal symptoms, social support, epistemic network analysis, semantic networks, hypergraphs, Reddit

## Abstract

**Background:**

Antidepressant use and withdrawal are often accompanied by side effects such as dizziness, weight gain, and sexual dysfunction. Antidepressants and their associated side effects are stigmatized topics. Social media platforms such as Reddit are considered “safe spaces” by users because they can freely share their experiences and receive support.

**Objective:**

This pilot study analyzed discussions from the subreddit r/depression to examine how users discuss antidepressant side effects, withdrawal symptoms, and related experiences of depression.

**Methods:**

We scraped 10 high-engagement threads from the subreddit r/depression using the Python wrapper for the Reddit application programming interface and conducted a 2-step analysis. First, a pilot test was performed using sertraline (Zoloft) threads, followed by an analysis of all antidepressant-related threads. A subset of the data was hand-coded to create and validate regular expressions, which were then used to automatically code the remaining dataset. The resulting coded data were analyzed using epistemic network analysis and complemented with qualitative analysis and elements of semantic networks and hypergraphs.

**Results:**

We found that posts were more likely to discuss emotional flattening, sleep, and memory or cognitive issues (Mann-Whitney *U*=33,235.5; *P*=.003). Additionally, references to dizziness tended to co-occur with discussions of withdrawal and offers of empathy, while reports of dream-related side effects and requests for personal experiences also co-occurred frequently. By incorporating elements of semantic networks and hypergraphs, we deduced that offers of empathy occurred when users said they experienced dizziness caused by withdrawal, while mentions of “brain zaps” associated with withdrawal often co-occurred with offers of teaching support.

**Conclusions:**

Study findings highlight how individuals experiencing antidepressant side effects and withdrawal symptoms use online forums such as Reddit to seek validation, share coping strategies, and provide emotional support to others. The nuanced discussions observed, particularly those related to empathy, symptom management, and shared learning, underscore the role of peer-to-peer networks in normalizing stigmatized experiences and mitigating isolation associated with antidepressant use. Clinicians and digital health practitioners can leverage these insights to better understand patient language, emotional framing, and informational needs outside clinical settings.

## Introduction

### Background

Depression is a mood disorder characterized by persistent sadness and loss of interest in daily life activities [1-3]. Each year, 1 in 10 adults in the United States experiences depression, and the incidence is approximately twice as high among women as among men [4,5]. To address this burden, antidepressants have become one of the most frequently prescribed medications, with approximately 10% of adults in the United States using them each year [2,6]. Although commonly associated with moderate to severe depression, antidepressants are also used to manage anxiety disorders and certain nonpsychiatric conditions [7].

Clinical guidelines, such as those from the American Psychological Association, recommend selective serotonin reuptake inhibitors (SSRIs) or other antidepressants as first-line treatments for adolescents and adults with depression [8]. Antidepressants fall into several classes, including SSRIs, serotonin-norepinephrine reuptake inhibitors, atypical antidepressants, serotonin modulators, tricyclic antidepressants, monoamine oxidase inhibitors, and N-methyl-D-aspartate antagonists. Among these, SSRIs such as sertraline and citalopram remain the most prescribed, along with amitriptyline, a tricyclic antidepressant [9].

Common adverse effects associated with antidepressant use include sexual dysfunction, weight gain, insomnia, and nausea [10-12]. Although these side effects often resolve with continued use, they can persist for some individuals. Additionally, people who have become accustomed to antidepressant exposure may experience withdrawal symptoms if they discontinue their medication. Common withdrawal effects include anxiety, worsened mood, and agitation [13]. These side effects, as well as depression and the use of antidepressants themselves, are stigmatized topics.

As the use of antidepressants increases, it is more critical than ever to move beyond simply identifying the side effects of antidepressants and instead leverage online forums to learn from the lived experiences of users, ultimately informing the development of more personalized therapies.

Social media platforms promote self-disclosure and disinhibition, in part because users can choose to remain anonymous or semianonymous [14]. Due to the high number and diversity of users, social media generates vast amounts of data, making it a valuable resource for research [15-17]. Reddit, for example, has more than 108 million daily active users, 100,000 user-created communities known as “subreddits,” and more than 22 billion posts and comments, making it a rich data source for discourse on stigmatized topics such as depression and peer support across diverse populations [18].

Reddit is increasingly used as a data source for qualitative and computational studies in public health and behavioral science. Researchers have used Reddit to investigate various topics, including COVID-19 vaccine side effects [17], vaping [16,19], depression and anxiety [20], loneliness [21], and substance use disorder [22]. Because users frequently share personal experiences and seek peer support, Reddit can offer unique insights into the lived experiences of hard-to-reach or underrepresented groups, including individuals using antidepressants.

### Social Support Behavior Code

Several studies examining the role of social support in online health discussions have used the Social Support Behavior Code (SSBC) to categorize the forms of support that social media users offer and receive from one another when discussing their health conditions [23-25]. SSBC includes informational, emotional, esteem, network, and tangible support types. Loans and expressions of willingness to perform tasks on behalf of another person are defined as tangible assistance, while compliments, offers of validation (through shared personal experiences), and attempts to relieve someone’s guilt are considered esteem support. Network support includes offers to “be there” for another person, access to companions, or reminders of existing supportive individuals. Informational support includes providing advice, offering factual information, re-evaluating situations, or making referrals. Finally, emotional support can include physical affection, such as hugs, sympathy, empathy, encouragement, prayers, and reminders of relational closeness with the person being supported. Improved understanding of how these support types are used in discussions of antidepressant side effects on Reddit could inform future studies on discussions of antidepressant use in health care settings.

### Epistemic Network Analysis

Epistemic network analysis (ENA) is a powerful analytical tool used to model relationships between ideas in qualitative data. Originally developed to study patterns of thinking and discourse in the learning sciences, ENA has since been applied across diverse domains, including health communication, education, and computational social science [26-30]. ENA quantifies and visualizes the relationships between concepts by mapping co-occurrences of codes within defined conversational or temporal windows. In an ENA network, these relationships are depicted through “network edges” that connect 2 nodes, with each node corresponding to a specific code that reflects a concept or theme within the dataset. A connection occurs when codes co-occur within a prespecified distance of each other. In a weighted ENA network, edge thickness, or weight, is proportional to the number of co-occurrences; therefore, a higher number of co-occurrences between 2 codes corresponds to a thicker edge between the nodes. ENA is unique because it goes beyond simple frequency counts to reveal how ideas are connected, making it useful for analyzing large textual datasets [26,27,31].

By focusing on the relationships between codes rather than isolated comments, ENA addresses a methodological gap by not only identifying key themes but also modeling the strength of the relationships between those themes. This approach offers a more nuanced understanding of how users construct meaning, express support, and share experiences, making ENA a useful tool for examining relationships among antidepressant use, side effects, withdrawal symptoms, and types of social support on Reddit. This pilot study aimed to examine how individuals discuss their use of antidepressants and experiences with withdrawal on Reddit by leveraging ENA to investigate the co-occurrence of key themes. Specifically, the objectives were as follows:

- To identify common antidepressant side effects, withdrawal experiences, and types of support expressed in online discussions of antidepressant use
- To visualize the relationships among common themes by constructing a network model
- To expand the conceptualization of edges within the ENA framework to capture the complexity of online discourse on antidepressant medications, reported side effects, withdrawal experiences, and support

## Methods

### Overview

Data were scraped from the subreddit r/depression using the Python (Python Software Foundation) wrapper for the Reddit application programming interface in February 2021. r/depression was chosen for this study because it has more members than other subreddits that discuss general antidepressant use. In total, 10 high-engagement threads were selected for analysis in this study. These threads were selected because they contained the highest number of comments, reflecting extensive discussion of antidepressant use, side effects, and withdrawal experiences. To better understand how users discuss their experiences with antidepressant use and withdrawal, we began by identifying the most frequently mentioned side effects across Reddit threads. Threads were included if they contained generic or brand names of drugs approved by the Food and Drug Administration to treat depression [32].

The *JMIR* digital ethics guideline was consulted when including Reddit posts, to ensure sensitivity to the protection of participants’ involvement in research conducted without direct consent [33].

Threads were first automatically separated into individual sentences, allowing coding to be performed at the sentence level [26,27]. Antidepressant generic and brand names were included as codes to allow identification of clusters of effects, symptoms, and 1 or more drugs. Other codes were developed through inductive and deductive coding. Coding was performed using the Reproducible Open Coding Kit (ROCK) web tool, iROCK, and the ROCK R package [3].

Refer to Multimedia Appendix 1 for the ENA computation steps. The semantic network diagram was created using qualitative network analysis (QNA) with the ROCK R package [34]. Hypergraphs were created manually. Decisions made on study methodology, data processing and management, and codebook development were recorded using the documentation standard Justifier [35].

### Ethical Considerations

The study was deemed exempt based on its nature. The University of Wisconsin-Madison provides a “Not Research Self-Certification Decision Tool,” which is available for not-research determinations. This tool was used in lieu of submitting an application to the institutional review board.

## Results

### Overview

Results revealed commonly discussed side effects that were consistent with prior literature, including emotional flattening, gastrointestinal distress, sexual dysfunction, sleep disturbances, and withdrawal symptoms (Table 1). The identified side effects were consistent with those commonly reported in previous studies [36-39].

**Table 1.** Codes, definitions, and representative quotes.

Name	Definition	Representative quotes
Depression	Depression, bipolar disorder, or premenstrual dysphoric disorder	“I never really had a sex drive for ages before (probably due to depression).”
Dose	Current dose or dose changes	“...a small dose won’t be as bad side effect wise.”
Emotional flattening	Apathy or feeling “numb” or “empty”	“Does anyone else on antidepressants feel numb that it’s almost impossible to cry?”
Gastrointestinal	Constipation, diarrhea, nausea, or vomiting	“Reacted very negatively to the Zoloft and had a couple days of vomiting.”
Memory and brain	Memory issues or “brain zaps”	“My own meds (an SSRI) cause memory loss...”
Physical pain	Headaches, migraines, or soreness	“The immediate side effects were the headaches.”
Sexual	Sexual dysfunction	“...must tell you that it kills your sex drive.”
Sleep	Changes in or issues with energy or sleep	“Taking the pills at night helps with the drowsiness.”
Weight	Changes in weight	“Anyway, I’m very apprehensive about the weight gain factor as a side effect.”
Withdrawal	Stopping medication	“...Pristiq is known for its horrible withdrawal...”

### Weighted ENA Network: Posts vs Comments

**Figure 1.**
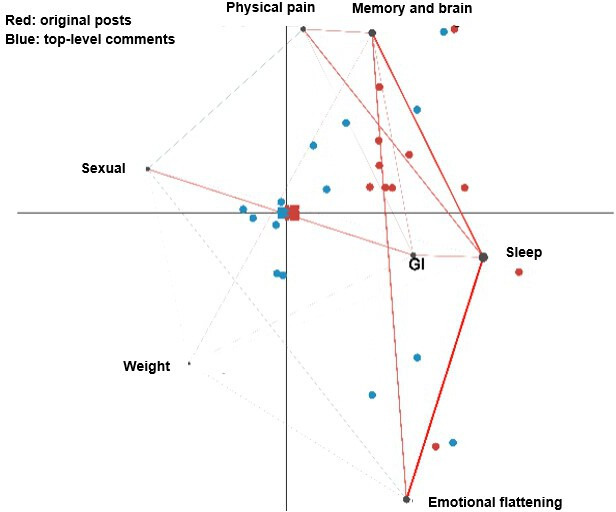
Weighted epistemic network analysis plot comparing original posts and top-level comments.

The weighted ENA revealed key structural differences between original posts and top-level comments (Figure 1).

Figure 1 shows the weighted network for posts (red) and top-level comments (blue) in the hand-coded test set. The groups were significantly different along the x-axis, suggesting that compared to top-level comments, posts were significantly more likely to discuss emotional flattening, sleep, and memory or brain issues (Mann-Whitney *U*=33,235.5; *P*=.003). Findings revealed frequent co-occurrences between withdrawal and symptoms such as emotional flattening and dream disturbances. Similarly, side effects such as memory issues, headaches, and changes in libido often appeared alongside discussions of dosage changes or discontinuation.

Clusters of data points indicate groups of posts and comments that warrant further analysis. Once these clusters are identified, a key analysis step will be to “close the interpretive loop.” This process will entail using the quantitatively identified data clusters as the starting point for a more in-depth, qualitative analysis of posts and comments in each cluster (Table 1). In this way, the network serves as an exploratory tool to identify patterns within a large, structurally complex dataset. Referring to the original data (“closing the interpretive loop”) allows interpretation of these co-occurrences.

### Patterns of Social Support in Side Effect Discourse

**Figure 2.**
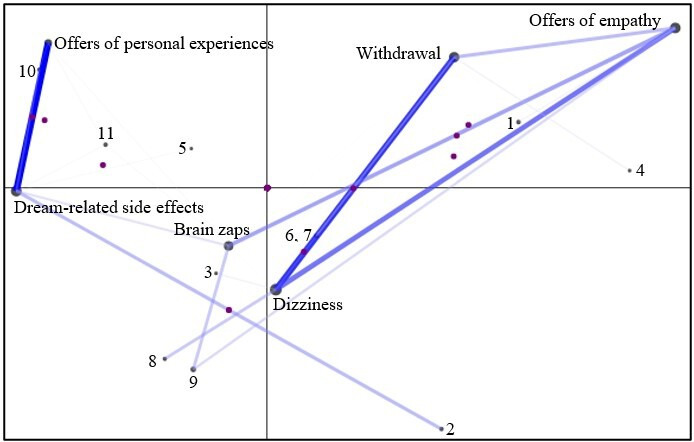
Weighted epistemic network analysis of social support types and antidepressant side effects. Violet-colored nodes labeled with words correspond to codes which occurred at least three times in the example thread. Darker and thicker lines indicate more frequent co-occurrences whereas lighter and thinner lines indicate fewer co-occurrences. Additional numbered codes infrequently occurred but are included here for comparison with alternative methods of data visualization represented in later figures (1=dependence, 2=requests for personal experiences, 3=offers of relief of blame, 4=vomiting, 5=offers of agreement, 6=offers of advice, 7=nausea, 8=requests for empathy, 9=offers of teaching, 10=requests for agreement, and 11=skipping or forgetting doses).

Analysis of co-occurring codes revealed distinct patterns of social support types associated with specific antidepressant side effects. For example, empathy and informational support frequently accompanied mentions of withdrawal and dizziness (Figure 2).

In the thread depicted in Figure 2, dark, thick lines indicate that offers of personal experience co-occurred with discussions of dreams. This relationship typically occurred when a user shared their experience with the side effect of altered dreams, as in the following example: “I have weird lucid [dreams] on fluoxetine...” References to dizziness also tended to occur with discussions of withdrawal and offers of empathy. Reports of dream-related side effects and requests for personal experiences also co-occurred frequently. Notably, offers of personal experiences frequently co-occurred with dream-related side effects. This finding suggests that when users request personal experiences regarding dream-related side effects, other users also share their own dream-related experiences.

Nodes labeled with words correspond to codes that occurred at least thrice in the example thread. Additional numbered codes occurred infrequently but are included here for comparison with alternative methods of data visualization represented in later figures (1=dependence, 2=requests for personal experiences, 3=offers of relief of blame, 4=vomiting, 5=offers of agreement, 6=offers of advice, 7=nausea, 8=requests for empathy, 9=offers of teaching, 10=requests for agreement, and 11=skipping or forgetting doses).

Although ENA provides a robust framework for modeling dyadic co-occurrences of concepts in Reddit posts, it captures only 1 layer of discourse structure. In this study, ENA was used to preserve sentence-level meaning by identifying which ideas were expressed together at the sentence level. However, ENA edges alone do not distinguish whether these co-occurrences reflect thematic overlap or if they also reflect causal attribution. They also do not capture higher-order configurations involving more than 2 concepts. Overall, ENA network edges depict co-occurrences of codes but do not contain any information about the type of relationship between codes, which is necessary for an in-depth analysis of Reddit data.

### Complementary Network Representations: Semantic Networks and Hypergraphs

To address the limitations of ENA, we suggest adding two features: (1) the freedom to note types of relationships between nodes (semantic relationships) and (2) the ability to connect more than 2 nodes (hypergraph elements).

For this study, we combined ENA with semantic network analysis and hypergraph modeling to capture different levels of antidepressant use discourse. ENA examined which ideas frequently appeared together within individual posts or sentences. Semantic network analysis identified the types of relationships between these ideas. Hypergraph modeling allowed us to interpret instances in which ≥3 ideas were expressed together in a single statement. There is substantial prior work on semantic networks and conceptual relationships. This work includes ConceptNet, a large semantic network and knowledge base [40], and the Unified Medical Language System semantic network, which identified 54 semantic links as part of a systematic effort to integrate multiple biomedical resources [1]. Previously identified connections include the “is a” relationship, which describes attributes such as types, conceptual containment, and generalization [2]. For instance, dizziness is a side effect of antidepressants. Additional relationships include causal (“produces” and “result of”), temporal (“occurs with” and “prerequisite event of”), spatial (“adjacent to” and “surrounds”), and functional (“prevents” and “motivation of”) links, among many others [41].

Examination of the aggregated semantic structure revealed that withdrawal-related symptoms appeared more frequently with emotional and informational support than did other side effects, suggesting a central role in support-seeking discourse. These observations informed the subsequent addition of hypergraph representations to explore higher-order meanings that were evident in the data but not fully captured by dyadic ENA edges alone.

**Figure 3.**
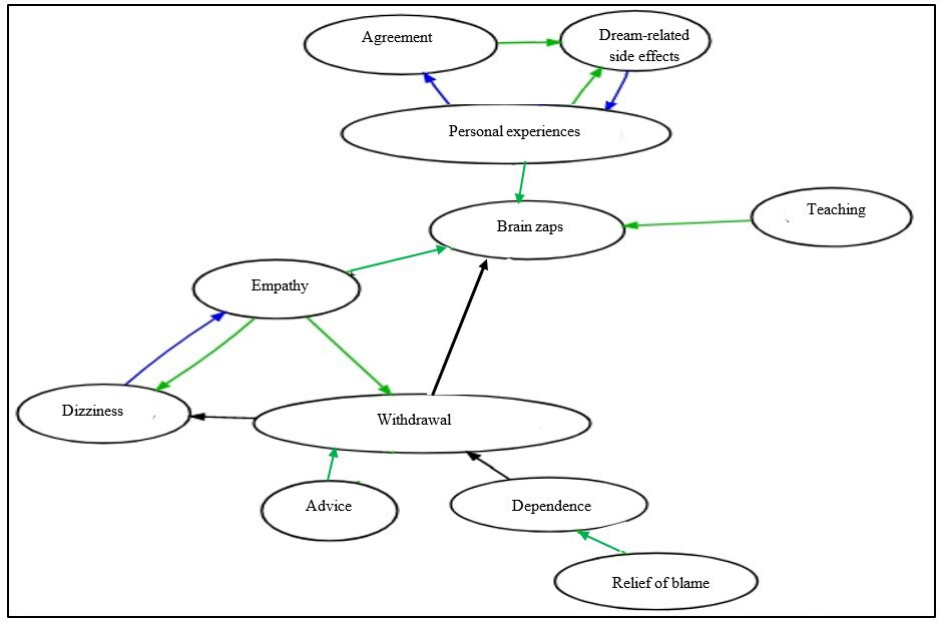
A qualitative network analysis representation of social support types and antidepressant side effects. Each node corresponds to a code. Arrows represent relationships between codes including causality (black), requests for support (blue), and offers of support (green).

Further exploration of the data using QNA revealed the need for more expressive representations. For example, although ENA clearly visualized pairwise relationships between codes, it became evident that some meaningful connections involved ≥3 concepts simultaneously. For example, in several threads, withdrawal, dizziness, and empathy were expressed together as part of a single experience. Incorporating hyperedges allowed us to represent these triadic relationships more precisely (Figure 3).

Similarly, semantic link types (eg, causal vs experiential) added interpretive depth, particularly when distinguishing between personal narratives and support offers.

To exemplify the utility of hyperedges, consider the following statement: *“*I went cold turkey and had the exact same situation. Vertigo to the point I was unable to leave the house for 3 days.*”* This statement offers empathy for dizziness caused by withdrawal. The connection among the 3 codes (empathy, dizziness, and withdrawal) is conceptually distinct from offering empathy for dizziness alone, as dizziness could be a side effect of antidepressant use, not associated with withdrawal. It is also different from offering empathy for withdrawal generally, as withdrawal often includes additional side effects. Although ENA would represent withdrawal-dizziness, dizziness-empathy, and withdrawal-empathy as 3 independent dyadic relationships, the hyperedge explicitly represents their co-occurrence. This distinction matters analytically because it differentiates empathy for an individual’s experience of depression or side effects from antidepressant use in general from empathy specifically tied to antidepressant withdrawal–related experiences.

Additionally, the ENA network contains 3 primary types of relationships: causality, support requests, and support offers. These relationship types and their associated directionality were captured using colored arrows in QNA.

**Figure 4.**
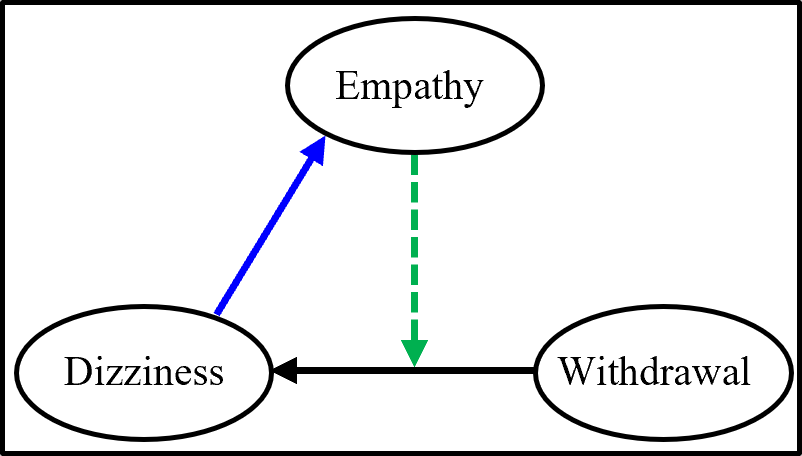
Modified QNA representation showing a relationship between more than two codes. In addition to a causal relationship between withdrawal and dizziness (black) and requests for empathy related to dizziness (blue), the example thread included offers of empathy for dizziness caused by withdrawal (green dashed).

Figure 4 shows a modified QNA representation with a hyperedge connecting 3 codes. In addition to a causal relationship between withdrawal and dizziness (black) and requests for empathy related to dizziness (blue), the example thread included offers of empathy for users who reported dizziness caused by antidepressant withdrawal (green dashed). QNA visualization allowed these patterns to be represented as directed and color-coded edges, clarifying the flow of discourse across codes.

The incorporation of semantic network properties and hyperedges into ENA raises issues related to network construction and interpretation. This study serves as a starting point for a rich discussion on the value of a multimethod approach for enhancing the depth and applicability of network analysis in the examination of social media discourse related to the use of antidepressants, side effects, and withdrawal experiences.

## Discussion

### Principal Findings

This pilot study examined how individuals discuss antidepressant side effects and withdrawal experiences on Reddit and how different forms of social support are embedded within these conversations. Using ENA complemented by semantic networks and hypergraph representations in QNA, we found that discussions of withdrawal-related symptoms, particularly dizziness and cognitive effects, frequently co-occurred with emotional and informational support. Posts describing withdrawal experiences tended to co-occur with offers of empathy and shared personal narratives, whereas other side effects were more commonly associated with informational exchanges. These patterns align with prior research showing that mental health discussions on Reddit are characterized by a high level of self-disclosure and active peer-to-peer support exchanges [14], and they extend established observations that online health communities often concentrate support around lived experience narratives and informational guidance [23-25]. The prominence of withdrawal-linked neurologic symptoms in our data is also consistent with clinical and patient-reported literature describing discontinuation effects such as dizziness and “brain zaps” as salient and, for some individuals, persistent [13]. At the same time, our finding that emotional flattening, sleep disruption, and memory or “brain” issues were especially prominent in original posts differs somewhat from that of large-scale social media work emphasizing sexual, weight, sleep, and pain-related side effect domains as major clusters [39]. This divergence may reflect differences in platform context, subreddit norms, sampling strategy (ie, high-engagement threads), and the analytic focus on co-occurrence structures rather than prevalence alone [39].

This study demonstrates the value of using ENA to explore complex discussions of antidepressant use, side effects, and withdrawal on Reddit. By modeling co-occurrences of themes and incorporating the SSBC, we identified meaningful patterns in how individuals seek and offer support around sensitive experiences with antidepressants. Our findings highlight not only the prevalence of commonly reported side effects, such as emotional flattening, dizziness, and sexual dysfunction, but also the layered ways in which these experiences are embedded in peer-to-peer support narratives on social media.

Although ENA proved effective for mapping conceptual relationships, our analysis also revealed its limitations when applied to large-scale, multidimensional social media data. Specifically, ENA’s pairwise edge structure can obscure more complex relationships involving multiple interrelated concepts or semantic links. To address this, we introduced the use of hyperedges and semantic networks within QNA to enhance the granularity and interpretive power of the network. Together, these methods offer a more nuanced lens for analyzing online health discourse and pave the way for future research integrating semantic meaning and structural complexity into digital health data analysis. Clinicians may use these findings to better understand patient language around withdrawal and side effects, thereby improving counseling and patient-centered care.

Still, Reddit users discussing antidepressants may not reflect the broader patient population. Useful extensions of this work may include cross-subreddit comparisons, temporal evolution of support patterns, or large-scale automated coding pipelines.

### Conclusions

Study findings can serve as a template for clinicians and digital health researchers seeking information about the types of support patients are already accessing online. Findings also highlight the need to complement, rather than replace, these peer-driven networks. Integrating these insights into clinical practice and digital intervention design may help bridge communication gaps, tailor patient education, and enhance trust by aligning clinical guidance with the real-world experiences expressed in online mental health communities.

## Supplementary material

10.2196/85812 Multimedia Appendix 1 Computation steps for epistemic network analyses.

## Abbreviations

ENA: epistemic network analysis
QNA: qualitative network analysis
ROCK: Reproducible Open Coding Kit
SSBC: Social Support Behavior Code
SSRI: selective serotonin reuptake inhibitor

## References

[1] Min H, Alemi F (2025). Insights into prescribing patterns for antidepressants: an evidence-based analysis. BMC Med Inform Decis Mak.

[2] de Sousa RD, Zagalo DM, Costa T, de Almeida JMC, Canhão H, Rodrigues A (2025). Exploring depression in adults over a decade: a review of longitudinal studies. BMC Psychiatry.

[3] Ormel J, Kessler RC, Schoevers R (2019). Depression: more treatment but no drop in prevalence: how effective is treatment? And can we do better?. Curr Opin Psychiatry.

[4] Simon GE, Moise N, Mohr DC (2024). Management of depression in adults: a review. JAMA.

[5] Goodwin RD, Dierker LC, Wu M, Galea S, Hoven CW, Weinberger AH (2022). Trends in U.S. depression prevalence from 2015 to 2020: the widening treatment gap. Am J Prev Med.

[6] Simon GE, Stewart C, Beck A (2014). National prevalence of receipt of antidepressant prescriptions by persons without a psychiatric diagnosis. Psychiatr Serv.

[7] Lunghi C, Dugas M, Leclerc J (2022). Global prevalence of antidepressant drug utilization in the community: protocol for a systematic review. BMJ Open.

[8] (2019). Clinical practice guideline for the treatment of depression across three age cohorts. https://www.apa.org/depression-guideline.

[9] Bogowicz P, Curtis HJ, Walker AJ, Cowen P, Geddes J, Goldacre B (2021). Trends and variation in antidepressant prescribing in English primary care: a retrospective longitudinal study. BJGP Open.

[10] Sheffler ZM, Patel P, Abdijadid S (2024). StatPearls.

[11] Wang SM, Han C, Bahk WM (2018). Addressing the side effects of contemporary antidepressant drugs: a comprehensive review. Chonnam Med J.

[12] Oliva V, Lippi M, Paci R (2021). Gastrointestinal side effects associated with antidepressant treatments in patients with major depressive disorder: a systematic review and meta-analysis. Prog Neuropsychopharmacol Biol Psychiatry.

[13] Horowitz MA, Buckman JE, Saunders R, Aguirre E, Davies J, Moncrieff J (2025). Antidepressants withdrawal effects and duration of use: a survey of patients enrolled in primary care psychotherapy services. Psychiatry Res.

[14] De Choudhury M, De S (2014). Mental health discourse on Reddit: self-disclosure, social support, and anonymity. Proc Int AAAI Conf Web Soc Media.

[15] Alambo A, Padhee S, Banerjee T, Thirunarayan K (2021). Pattern Recognition. ICPR International Workshops and Challenges.

[16] Chen AT, Zhu SH, Conway M (2015). What online communities can tell us about electronic cigarettes and hookah use: a study using text mining and visualization techniques. J Med Internet Res.

[17] Kwon S, Park A (2023). Examining thematic and emotional differences across Twitter, Reddit, and YouTube: the case of COVID-19 vaccine side effects. Comput Human Behav.

[18] Press - Reddit. Reddit.

[19] Wu D, Kasson E, Singh AK (2022). Topics and sentiment surrounding vaping on Twitter and Reddit during the 2019 e-cigarette and vaping use-associated lung injury outbreak: comparative study. J Med Internet Res.

[20] Boettcher N (2021). Studies of depression and anxiety using Reddit as a data source: scoping review. JMIR Ment Health.

[21] Shah HA, Househ M (2025). Understanding loneliness through analysis of Twitter and Reddit data: comparative study. Interact J Med Res.

[22] Chi Y, Chen HY (2023). Investigating substance use via Reddit: systematic scoping review. J Med Internet Res.

[23] Coursaris CK, Liu M (2009). An analysis of social support exchanges in online HIV/AIDS self-help groups. Comput Hum Behav.

[24] Bronstein J (2017). An examination of social and informational support behavior codes on the internet: the case of online health communities. Libr Inf Sci Res.

[25] Atwood ME, Friedman A, Meisner BA, Cassin SE (2018). The exchange of social support on online bariatric surgery discussion forums: a mixed-methods content analysis. Health Commun.

[26] Shaffer DW, Collier W, Ruis AR (2016). A tutorial on epistemic network analysis: analyzing the structure of connections in cognitive, social, and interaction data. J Learn Anal.

[27] Shaffer DW (2017). Quantitative Ethnography.

[28] nCoder. https://www.n-coder.org/.

[29] Choi J, Cai Z, Marquart C, Ruis AR, Shaffer DW, Wasson B, Zörgő S (2021). Third International Conference on Quantitative Ethnography: Conference Proceedings Supplement.

[30] Marquart CL, Muhammad HA, Shaffer DW Cran: package tma.

[31] Zörgő S, Peters GJ (2019). International Conference on Quantitative Ethnography.

[32] Depression medicines: from the FDA office of women’s health. U.S. Food and Drug Administration.

[33] Herington J, Connelly K, Illes J (2023). Ethical imperatives for working with diverse populations in digital research. J Med Internet Res.

[34] Peters GJ, Zorgo S Reproducible open coding kit. Rock.

[35] Peters GJ Human and machine-readable justifications and justified decisions based on ‘YAML’. GitHub.

[36] Cascade E, Kalali AH, Kennedy SH (2009). Real-world data on SSRI antidepressant side effects. Psychiatry (Edgmont).

[37] Anderson C, Roy T (2013). Patient experiences of taking antidepressants for depression: a secondary qualitative analysis. Res Social Adm Pharm.

[38] Anderson HD, Pace WD, Libby AM, West DR, Valuck RJ (2012). Rates of 5 common antidepressant side effects among new adult and adolescent cases of depression: a retrospective US claims study. Clin Ther.

[39] Saha K, Torous J, Kiciman E, De Choudhury M (2021). Understanding side effects of antidepressants: large-scale longitudinal study on social media data. JMIR Ment Health.

[40] Liu H, Singh P (2004). ConceptNet — a practical commonsense reasoning tool-kit. BT Technol J.

[41] Brachman RJ (1983). What IS-A is and isn’t: an analysis of taxonomic links in semantic networks. Computer.

