# Supplementary material for: Mapping Peer Support in Antidepressant Discussions on Reddit: Pilot Network Analysis
Source: J Particip Med. 2026 Jun 12;18:e85812. doi: 10.2196/85812 (PMC13263020; doi:10.2196/85812)
Supplement: Multimedia Appendix 1 [file jopm-v18-e85812-s001.docx]

**Appendix 1: ENA Computation Steps**


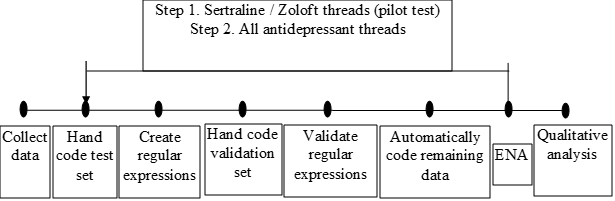


To develop regular expressions for eventually automatically coding the full data set, a subset of threads with posts containing “sertraline” (53, or 1.96%) and those containing “Zoloft” (137, or 1.81%) was used as a test set. A validation set was created using another 79 (2.91%) “sertraline” threads and 226 (2.99%) “Zoloft” threads. The test and validation sets were coded manually. The inter-rater reliability was > 0.85 and Shaffer’s rho was < 0.05. Using the regular expressions, data was automatically coded using the R package nCodeR.
